# Lateral river erosion impacts the preservation of Neolithic enclosures in alluvial plains

**DOI:** 10.1038/s41598-023-43849-6

**Published:** 2023-10-02

**Authors:** Jean-Louis Grimaud, Patrick Gouge, Damien Huyghe, Christophe Petit, Laurence Lestel, David Eschbach, Martin Lemay, Jean Catry, Ibtissem Quaisse, Amélie Imperor, Léo Szewczyk, Daniel Mordant

**Affiliations:** 1 PSL University/MINES Paris/Centre de Géosciences, 35 rue St Honoré, 77305 Fontainebleau Cedex, France; 2 Département de Seine-et-Marne/Centre departemental d’archéologie de la Bassée, 11 rue des Roises, 77118 Bazoches-lès-Bray, France; 3 Université Paris 1 Panthéon-Sorbonne, UMR 7041 ArScAn, 6, rue Michelet, 75006 Paris, France; 4 Sorbonne Université, CNRS, EPHE, UMR 7619 Metis, 4 place Jussieu, 75005 Paris, France; 5 Akkodis, 4 rue Jules Ferry, 64000 Pau, France

**Keywords:** Geomorphology, Archaeology

## Abstract

Situating prehistoric sites in their past environment helps us to understand their functionality and the organization of early sedentary human societies. However, this is a challenge as the natural environment constantly evolves through time and erases these constructions, especially along riverbanks, thus biasing the archaeological record. This study introduces a reassessment of the paleo-landscape evolution around the Neolithic enclosures at the Noyen-sur-Seine site based on new field observations as well as the synthesis of (un)published and new radiocarbon dating. Contrary to the initial hypothesis, our results show that the Noyen enclosures were not built along a Neolithic Seine River: the nearby channels were active in the Middle Age and Early Modern periods. Therefore, the results show that the enclosures were originally much larger: only a fraction that survived river erosion (lateral migration rates up to 2–3 m yr^−1^ estimated during the nineteenth century) has been preserved. Instead, an abandoned Mesolithic Seine River served as a natural delimitation of the SE part of the Neolithic enclosures. These results indicate that Neolithic enclosures in alluvial settings are often only partly preserved and that societies from that period lived farther away from active rivers than originally thought, where they were protected from floods.

## Introduction

Appreciating alluvial landscape evolution in the vicinity of archaeological sites is a prerequisite to better understand the function(s) and significance of settlements and edifices that have been abandoned there for centuries. This is particularly necessary for Prehistoric periods when humans did not leave any figurative representation of their environment. Despite their ubiquity and sometimes monumental size (i.e., associated ditches were sometimes up to 7 m wide^1^), the function(s) of Neolithic enclosures is still a matter of discussion^2–5^. To date, paleo-landscape or paleo-environment reconstructions of the Neolithic enclosures have often been proposed based on their modern geography. This is always an oversimplification: natural environments and landscapes must be considered as evolving at the centennial to millennial scale, and the associated archaeological sites are subject to erosion^6^. The configuration of rivers and waterways is particularly mobile^7–10^ and can be modified by their internal dynamics but also by climate variations and human activities^11^. For example, recent geoarchaeological investigations allowed researchers to revise existing interpretations of the major archaeological site of Chauvet Cave^12^ by specifying the timing of the evolution (incision and cutoffs) of the neighboring Ardèche River.

Alluvial domains are propitious to settlement owing to the accessibility of water resources. In these areas, river paths change at the centennial-millennial scale with channel avulsions (i.e., the displacement of a river section to a new path) and at the decennial scale with channel lateral erosion^13,14^. As rivers migrate, the sedimentary record in the alluvial plain is reworked^15,16^. Erosion is also likely to affect the archaeological record, which is highly incomplete, similarly to the underlying geologic record^17^. Due to this preservation bias, the perception of archaeological sites in their current landscape may be different from their original configurations. In this case, a careful reconstitution of the landscape evolution dynamics based on geomorphic and sedimentary analyses is necessary before proceeding to further interpretations of the dynamics of early settlement.

**Figure 1:**
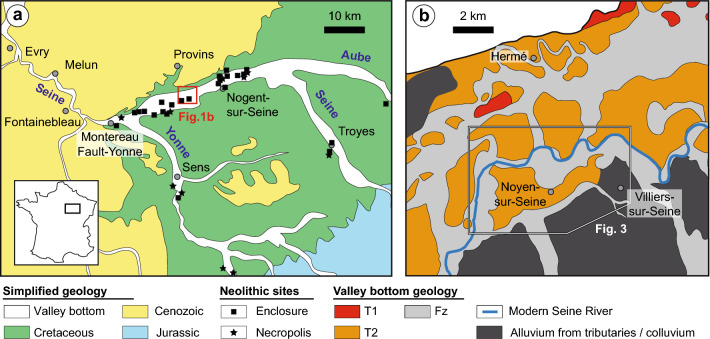
Location of the study area. (**a**). Geological map^55^ showing the valley bottoms together with the main enclosures and necropolises from the Neolithic period^5^. La Bassée, is an alluvial plain of the Seine River upstream of Paris, but downstream of the Aube River and upstream of the Yonne River. It is parallel to the cuesta limiting the Cretaceous chalk and the Tertiary. (**b**). Details of the geology of the valley bottom showing the Quaternary surface formations^23^.

**Figure 2:**
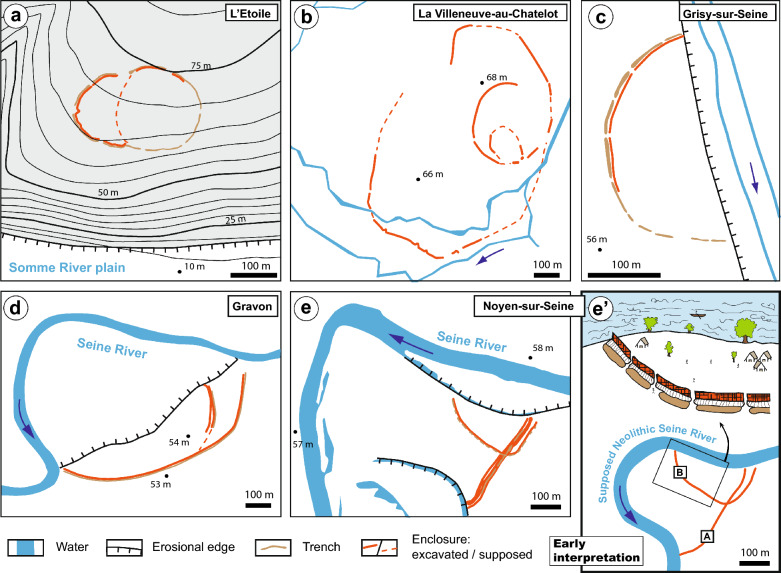
Examples of non-alluvial (**a**) and alluvial (**b**–**e**) Neolithic enclosures found in northern France^1,48–50^. Note that (**a**) and (**b**) have a circular shape and that the limit of (**c**), (**d**) and (**e**) correspond to erosional boundaries. (**e′**) early reconstruction of the Noyen-sur-Seine landscape. For example, it is assumed that the B enclosure was delimited by a Neolithic Seine River, which implied the implantation of early sedentary societies by the river. In this study, this model is challenged by showing that the erosional boundaries occurred in Noyen-sur-Seine much later than the Neolithic.

There are several stages of human settlements known during the Neolithic period in NW Europe (6000–2200 yrs BC)^18,19^. To better understand the conditions of these settlement stages, special attention must be paid to the paleoenvironmental context of the Neolithic sites. The floodplains of the Paris Basin—especially along the Seine River—contain numerous archaeological features from this period (pit or trench, semicircular enclosures, burial sites etc.), which are often excavated as a part of planned research or preventive surveys^20,21^ (Figs. 1a and 2). The part of the alluvial plain between Conflans-sur-Seine and Montereau-Fault-Yonne, France, which is called *La Bassée*, has been intensely researched since the early 60’s primarily due to the development of preventive archeology associated with the opening of gravel pits. The plain was shaped by river incisions and subsequent alluvial depositions during the last two glacial periods^22^. Many deposits from the Late Glacial Maximum until the Present are found in the form of multiple channels, levees (embankment), and overbank flood and peatland deposits^23–27^. Prehistoric remnants are found in association with these deposits.

**Figure 3:**
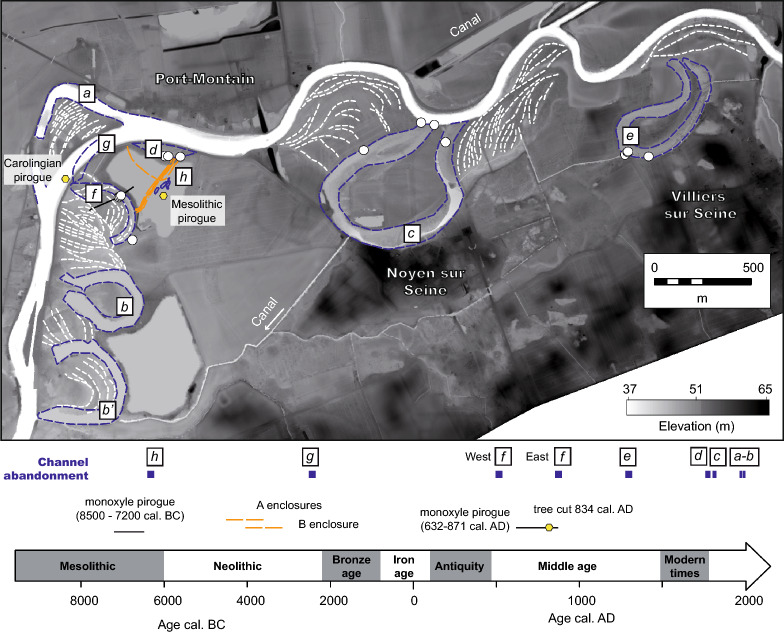
Geomorphic map of the study area, showing paleochannels (dashed blue lines), crescent bars (dashed white lines) and Neolithic enclosures (orange dashed lines). A chronology with the relative and absolute dating of channels a to h, as well as the two monoxyle pirogues are presented. The white dots correspond to locations where field observations and/or radiocarbon sampling were performed.

In the Bassée area, aerial photographs revealed a large-scale enclosure system at Noyen-sur-Seine (a place called Le Haut des Nachères)^28^ bounded by several abandoned meanders defining define paleo-locations of the Seine River (Figs. 2 and 3). Some of these channels had been abandoned recently^22^ (between the nineteenth century and the present day) but are still hydraulically connected to the Seine River (i.e., channels a, b and c; Fig. 3). The timing of the abandonment of the other channels is not well known. The discovery of a large quantity of pottery sherds and flint from the Middle Neolithic on the site’s surface^29^ led to the beginning of archaeological research and the systematic mapping of the enclosures system in 1972. Enclosure A (Fig. 2e), orientated roughly NE-SW, is composed of series of sub-parallel palisades and trenches. Enclosure B has a curved shape and is surrounded by a trench that is much deeper than the ones associated with enclosure A. Radiocarbon dating of faunal bones found within the different trenches yielded ages for enclosures A (4350–3100 yrs cal. BC) and B (4000–3350 yrs cal. BC) (Table 1). In 1983, artefacts were collected in the alluvium and peat at the base of a clogged channel located SE of the Neolithic enclosures (faunal remains, flint flakes, flint tools, fragments of fish traps and a pirogue) and dated as belonging to the Mesolithic period. In 1992, earthwork to straighten the meander of the Seine to the north and west of the Neolithic site led to the unexpected discovery of a second pirogue; but this one has been dated to the beginning of the Middle Age period, more precisely the Carolingian period^20,30^. Early models suggest that the Neolithic enclosures were partly built in the vicinity of—and delimitated by—the Seine River^29^ (Fig. 2e′). This theory was based on the occurrence of two channels delimitating the site to the SE and to the NW of the archaeological site (channels d and f; Figs. 2e and 3), and that were thought to be Neolithic in age^31–33^. This interpretation taken for granted^34^ thus envisaged a society well adapted to its environment, taking advantage of the natural delimitation that the Seine River may have provided.

**Table 1:** Radiocarbon dating.

Sample reference	Location	Material	Age BP	± Δ	*Terminus post quem (Age cal AD)*	*Terminus ante quem (Age cal. AD)*	Period
Gif-7286	Channel system h	Wood	9130	± 100	− 8650	− 7950	Mesolithic
Gif-6632	Channel system h	Wood (trunk)	8020	± 100	− 7350	− 6600	
Gif-6633	Channel system h	Wood (fish trap)	8000	± 100	− 7300	− 6600	
Gif-6631	Channel system h	Wood (stack)	7990	± 100	− 7300	− 6550	
Gif-6559	Channel system h	Wood (pirogue)	7960	± 100	− 7200	− 6500	
Gif-6989	Channel system h	Wood (plank)	7400	± 80	− 6430	− 6070	
Gif-7126	Channel system h	Wood (branch)	7300	± 80	− 6380	− 5990	
Gif-7125	Channel system h	Wood (bark)	7040	± 80	− 6030	− 5730	
Gif-6991	Channel system h	Wood (branch)	6240	± 70	− 5370	− 4990	Neolithic
Gif-6043	Channel system h	Wood (oak piece)	5800	± 80	− 4810	− 4450	
Gif-6990	Channel system h	Wood	5400	± 70	− 4360	− 4040	
Ly-2462	Enclosure A	Fauna bone	5060	± 170	− 4350	− 3500	
Gif-7285	Channel system h	Wood	4960	± 70	− 3950	− 3640	
Ly-2461	Enclosure B	Fauna bone	4890	± 140	− 4000	− 3350	
Ly-2457	Enclosure A	Fauna bone	4870	± 160	− 4050	− 3100	
Ly-6064	Channel g	Wood (trunk)	3782	± 53	− 2410	− 2030	Bronze age
Ly-6066	Channel g	Wood (trunk)	3696	± 53	− 2280	− 1920	
Ly-6065	Channel g	Wood (oak piece)	3197	± 52	− 1610	− 1310	
Ly-6063	Channel f west	Wood (plank)	1731	± 49	130	430	Antiquity
Ly-6060	Channel f west	Wood (stack)	1717	± 48	210	430	
Beta-589086	Channel f east	Plant	1490	± 30	545	642	Middle age
Beta-589085	Channel f east	Organic sediment	1280	± 30	662	774	
Ly-5891	Channel f west	Wood (pirogue)	1304	± 64	580	970	
Ly-6062	Channel f west	Wood (clumps)	1289	± 50	650	880	
Ly-6059	Channel f west	Wood (stack)	1152	± 48	770	1000	
Ly-6061	Channel f west	Wood (stick)	1055	± 55	870	1160	
Poz-154562	Channel e	Organic sediment	610	± 30	1299	1404	
Poz-154599	Channel d	Organic sediment	40	± 30	1811	1917	Modern times

Therefore, hypotheses on the age of channels at Noyen-sur-Seine were behind the suggestion that a first Neolithic enclosure took advantage of the meander as a natural delimitation and that a second, later Neolithic enclosure was built along the Seine River. However, the age of these channels was never determined, and the early interpretation remained entrenched in the community. A reassessment of the chronologies of channels migration and abandonment seems therefore necessary. In this study, we provide these estimates, both directly—by dating the organic infill of the abandoned using radiocarbon techniques—and indirectly—by assessing the lateral migration rate and paths of the paleo-Seine River based on the analysis of historical maps. We show that the Neolithic configuration of the site was different than originally thought and we discuss the broader implications for the location and size of enclosures in alluvial settings.

## Results and discussion

### Historical rates of river migration

**Figure 4:**
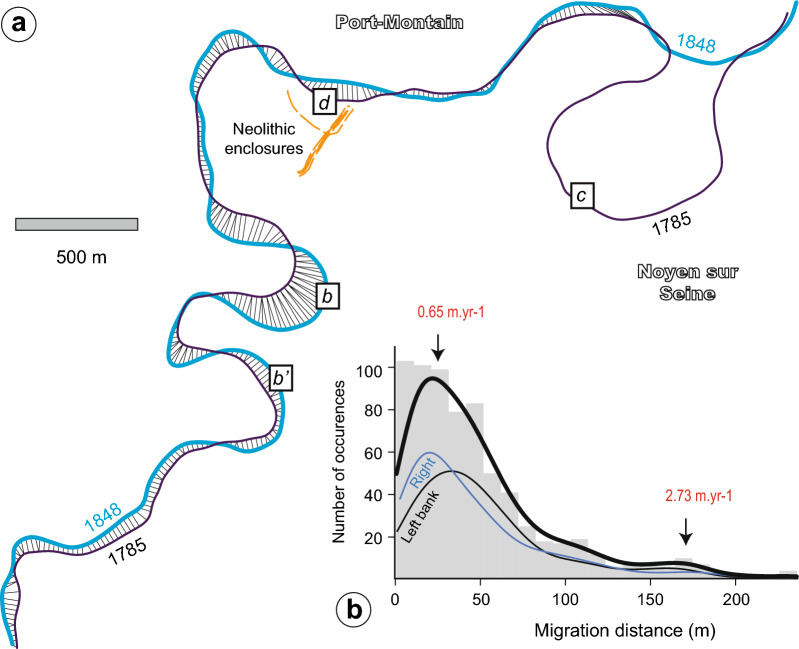
Reconstruction of the lateral rates of migration from historical maps (**a**) Example of the migration distance calculated using the the dynamic time warping (DTW) algorithm^36^ for the left bank of the Seine River in 1785 and 1848. The letters b, c and d correspond to the paleochannels located in Fig. 3. (**b**) Distribution of the estimated migration distance for the left bank (thin black curve) and right bank (thin blue curve) as well as the total (thick black curve). Some corresponding lateral migration rates are indicated in red. The migration rates are calculated by dividing the distance by the time between maps (i.e., 63 yrs).

In the Bassée alluvial plain, the modern Seine River has been completely canalized between artificial riverbanks since the mid-nineteenth century, which prevents river migration (Fig. 3). The purpose of these intense human-induced modifications of the Seine River—shown by historical maps^35^—was to improve navigation. Two historic maps of the area near Noyen-sur-Seine (Fig. 4), dating from 1785 and 1848, were used (see Supplementary Materials) to measure the lateral migration rates of the river. The maps were digitized to generate and compare the 1785 and 1848 banks along a ca. 8–10 km reach using a dynamic time warping algorithm^36–38^ (see “Methodology” section). The estimated uncertainties for river path location was 18 m in average, which corresponds to a migration rate of 0.33 m yr^−1^. The results yielded migration rates with median values of 0.65 and maximum of 2.73 m yr^−1^. The minimum values corresponded to straight sections and the maximum values to curved sections (Fig. 4)^39^. These values are higher than the estimated uncertainties. The measured rates are within the range of known values for the lateral migration of vegetated rivers^40–42^.

It is always challenging to extrapolate the calculated migration rates from the Early Modern period further back in time given that the measured migration rates depend on the timescale for the river migration^40^ and, above all, because the configuration of the river pattern was different in the La Bassée area^43,44^. Thus, another estimation of the migration rate was obtained based on the trajectory of the Seine River after a neck cutoff in the Villiers-sur Seine area (channel e; Fig. 3). Organic sediment at the bottom (1.25 m deep) of the fine-grained infill within the abandoned e channel (Fig. 3) gave a minimum radiocarbon age of 610 ± 30 yrs BP (i.e., 1299–1404 yrs cal. AD) for this cutoff event. Since the cutoff, the Seine River migrated up to 200 m towards the North, based on the distance between the neck of the abandoned channel and the current position of the Seine River (Fig. 3). By dividing this distance by the time since abandonment, a maximum migration range of ca. 0.33 m yr^−1^ can be estimated. This other result therefore confirms the continuity of the migration rates for centuries, although with a lower value. Hence, the historic Seine River had the potential to migrate up to several hundred meters. In this case, it seems very unlikely that the large Neolithic enclosures built along the riverbanks were completely preserved—such as suggested in Noyen-sur-Seine^32^. It seems more plausible that they would have been at least partly eroded.

### A historical origin of the channels adjacent to the Neolithic enclosures

**Figure 5:**
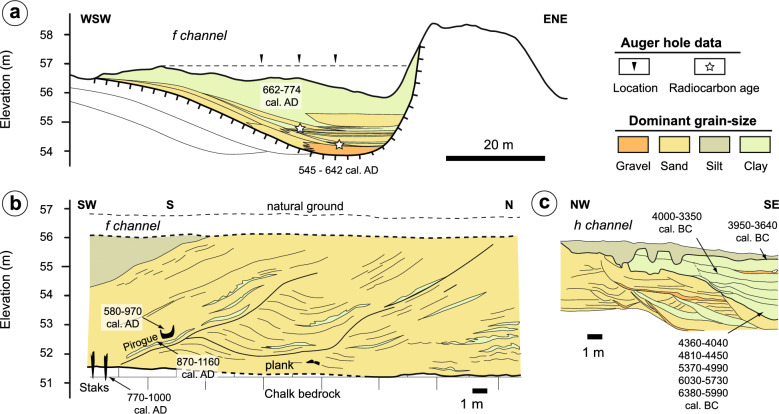
Sedimentary cross-sections from the abandoned channels with the reported radiocarbon dates: (**a**) east f channel, (**b**) west f channel, (**c**) h channel. See Fig. 6 for locations. Cross-section b is from Mordant et al.^30^.

**Figure 6:**
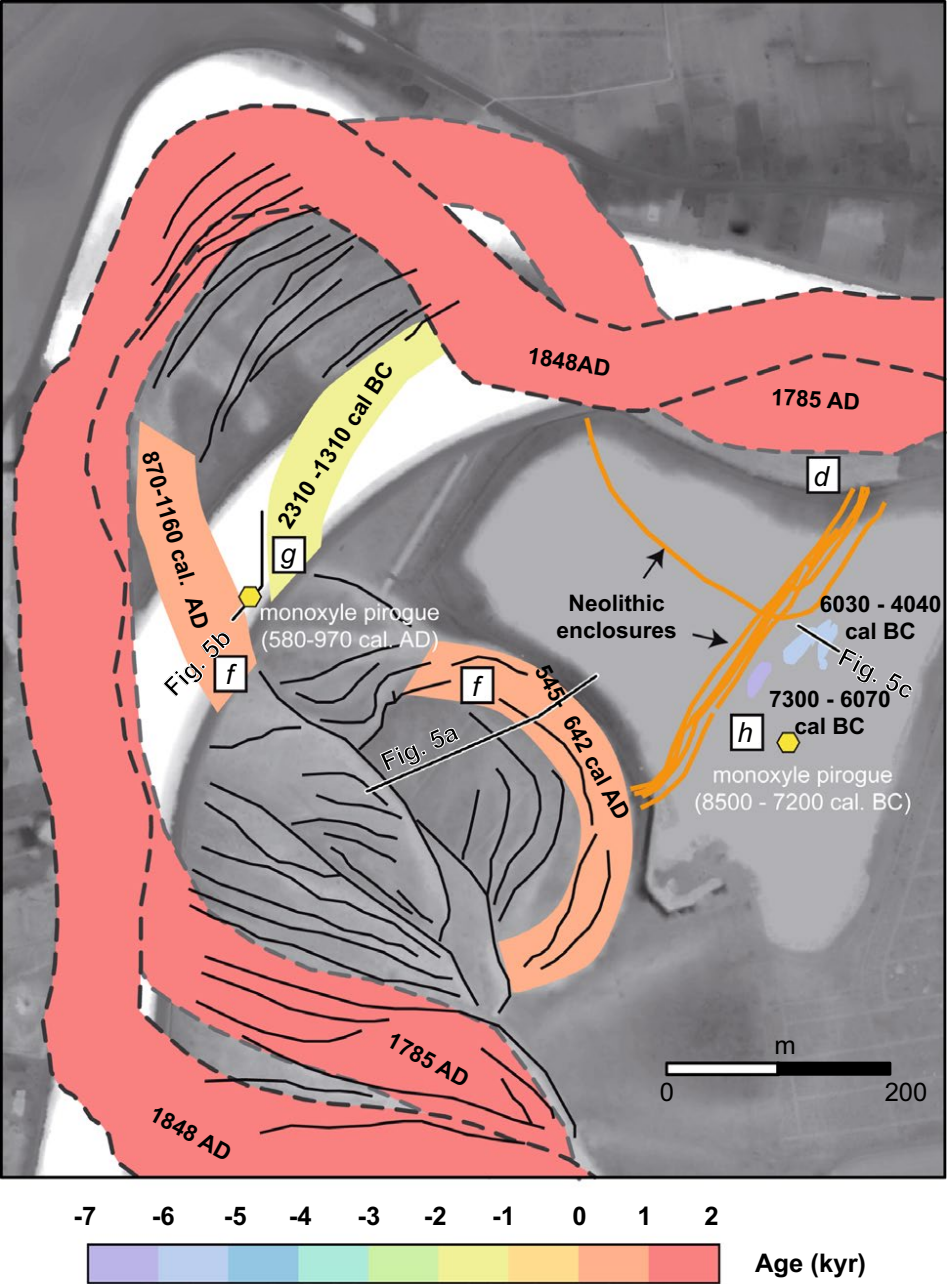
Superimposed paths of the channels based on geoarchaeological mapping and historical maps (see Supplementary Materials). The location of the cross-sections in Fig. 5 is reported. The letters d, f, g and h correspond to the paleochannels shown in Fig. 3.

To go further back in time, a chronological analysis of the abandoned channels near Noyen-sur-Seine and Villiers-sur-Seine was performed from the data available in publications and unpublished reports, as well as new samplings and radiocarbon dating^30^ (Figs. 3, 5 and 6) (Table 1). Several groups of channels were identified.

In the studied area, a first group of historic channel abandonments (cutoffs) is related to modifications made by humans for navigation purposes in the years 1992 (channel a), 1855 (channels b and b’) and 1809 (channel c)^44,45^. The abandonment of channel c is visible between the 1785 and 1848 maps, confirming its historical origin (Figs. 3 and 5). The trace of channel d, located directly to the north of the Neolithic enclosure is visible on the 1785 map (Figs. 3 and 6). Contrary to the earlier view that assumed a Neolithic age for this channel^31^, this indicates a recent origin. A radiocarbon age calculated on a 40 cm deep sample within the channel d sediment returned a date of 1811–1917 yrs cal. AD. Hence, historical maps and radiocarbon dating of the sediment infill of the abandoned channels demonstrated that the abandonment of the channel to the north of the enclosure is much more recent than the Neolithic period. Therefore, the northern d channel was never a boundary for the Neolithic enclosure as it was abandoned much more recently (i.e., less than 150 years ago).

Another group of channels from the Middle Age period (e and f) is identified (Fig. 3). Channel f, located to the southwest of the Neolithic enclosure, has been studied in particular. It was considered as part of the hypothetical Neolithic Seine River in early reconstructions^31^ along with channel d. The latter is shown above to be very recent. To the west of this channel, the Carolingian pirogue was found during canal digging in 1992^46^. The proximity of the pirogue and the channel suggests that they are chronologically close, which is confirmed by the sedimentological record and radiochronology (Fig. 5). An early phase of disconnection of channel f started around 545–642 yrs cal. AD in its eastern part (Fig. 5a), in association with relatively coarse filling. Wood artifacts (i.e., the pirogue, planks and shafts directly stuck in the underlying chalk bedrock) highlight that the river activity lasted a bit longer in the western part of the channel. These observations point to a cutoff event between 550 and 650 yrs cal. AD, which isolated the eastern part of the channel, likely through the formation of a sand plug blocking the entrance^47^. This cutoff was followed by the lateral migration of the Seine River towards the west, which enabled the Carolingian pirogue to become stranded after ca. 850 yrs cal. AD. The results therefore invalidate the conclusion of a Neolithic origin for the southwestern f channel, similarly to the northern channel d (Fig. 3).

Archaeological investigations carried out in 1992 revealed numerous other features of the channel, which could be dated (Fig. 5a–c). For instance, a series of trunks was found in channel g and consistently gave radiocarbon ages from the Bronze Age (Fig. 3, Table 1). Other remnants from Antiquity were found in the vicinity of channel f. It was difficult to evaluate whether these remains were found in place on the site or if they were transported by riverine or human activity. Lastly, a series of channel pools partly filled with organic sediment—which are loosely defined as the channel h—were investigated by preventive archaeological projects in the 1980’s^20^. An unpublished cross-section of the channel based on these investigations is shown in Fig. 5c. The cross-section indicates a complex sedimentary fill that started at the limit between the Mesolithic and Neolithic periods around 6300 BC. It is important to note that the channel pool was filled with 2–3 m of sediment until it reached a level close to that of the current naturel ground by 3600 BC, i.e., at the time of Neolithic enclosures. Thus, this paleochannel (h; Fig. 3), whose bankfull activity dated back to the Mesolithic, was much shallower during the Neolithic period. Once again, no bankfull active Neolithic Seine River could be identified in the vicinity of the Neolithic enclosures.

### Middle to Late Holocene landscape evolution

Figure 6 summarizes the mapping of the channel features by time periods in the vicinity of the Neolithic enclosures. The Mesolithic and Neolithic channels have very small extensions compared to the most recent ones. To better reconstruct the motions of the channel between stages, crescent bars at the top of point bar deposits were mapped (Figs. 3 and 6). They made it possible to track the successive positions of the river channel banks during their lateral displacement. For instance, along the cross-section presented in Fig. 5a, it is possible to infer 200 m of motion for the f channel, which helped erode the southwestern part of the enclosure. Considering—from the position of the Bronze Age (g) channel—that this eastward motion occurred after 1500 yr cal. BC, a lateral migration rate for the river of 0.1 m yr^−1^ can be estimated. This rate must be considered as a minimum given that a hiatus is observed during the Iron Age: no radiocarbons from that period are found in the study area^30^. Similarly, it is possible to estimate the southward lateral migration rate of the Seine River after the cutoff event from ca. 540 to 1785 yrs cal. AD to be on the order of 0.2 m yr^−1^. The two values are relatively close and consistent with modern and centennial estimates. They further indicate that the d and f channels were eroded over a distance of at least several hundreds of meters along the enclosure perimeter before being abandoned.

**Figure 7:**
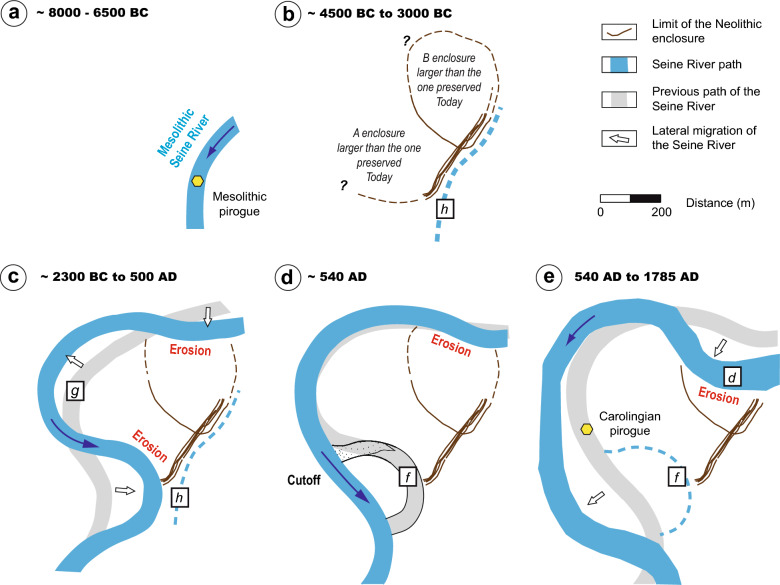
Reconstructed evolution of the landscape since the Mesolithic showing the progressive erosion of the Neolithic enclosures due to the migration of the Seine River. The reconstruction supports the hypothesis that a system of large, circular enclosures was progressively eroded along the bank of the laterally migrating Seine River. The locus where the two pirogues were abandoned is also reported.

Together, the direct and indirect estimates for the migration of the Seine River in the vicinity of Noyen-sur-Seine can be used to reconstruct the evolution of the natural environment and landscape since the Mesolithic period (Fig. 7). The Mesolithic pirogue was found in a sandy layer not far from the channel pools filled with organic sediment. This suggests the proximity of an active channel of the Mesolithic Seine River (Fig. 7a). We can assume that this channel was abandoned around 7300 yr BC, based on the age of the artefacts found in the peat at the base of the channel fill (Fig. 5c, Table 1). A first phase of filling for the h channel—mostly with fine sediment—lasted until ca. 4000 yr BC, based on evidences of trampling and combustion found in the associated sedimentary level. The Neolithic enclosure of Noyen-sur-Seine was built during the same period (ca. 4500–3000 yrs BC; Fig. 6b). The remarkable preservation of the enclosure near the h channel supports the hypothesis that the abandoned channel served as a natural delimitation^20^—a sort of ditch—that could be crossed by foot. From 2300 yr BC to the present, the migration of the Seine River triggered bank erosion, which led to the removal of parts of the enclosure, particularly during the late Carolingian period and Early Modern period (Fig. 7c–e).

From ca. 6300 to 2300 yrs BC, the Seine River cannot be precisely located in the study area. The progressive filling of the Mesolithic h channel with sediment during the Neolithic likely attests that no reactivation of this channel occurred as long as the enclosures were established in the area. The active Seine River was therefore positioned away from the site, probably farther north: Geological mapping indeed indicates that another channel belt exists near a village called Hermé (Fig. 1b). It was demonstrated earlier that the hypothetical candidates for the Neolithic Seine (i.e., the d and f channels) were in fact historically abandoned channels. Here, the data indicate that the enclosures were nowhere near the Seine River during the Neolithic. As in other alluvial plains, the geography of La Bassée changed as the Seine River changed its course^43,44^, i.e., with an avulsion. During the Neolithic period, while the river occupied other areas, abandoned channel corridors (i.e., near the h channel) were therefore more likely to be occupied as shown by the enclosures of Noyen-sur-Seine.

### Reconsiderations for Neolithic enclosures

The demonstrations that the nearby f and d channels were respectively abandoned in the Middle Age and Early Modern periods show that the Noyen-sur-Seine enclosures were not built along a Neolithic Seine River as originally thought. In addition, the reconstruction of the river migration path indicates significant loss of the area delimited by the two enclosures over the past 4300 years due to fluvial erosion (Fig. 7). The enclosures were therefore originally much larger than what is preserved today (ca. 900 m long and 25,000 m^2^ for the inner one and 1800 m long 70,000–80,000 m^2^ for the outer one). Thus, the currently measured dimensions of the enclosures are the result of having been preserved from erosion due to river activity. These dimensions have relatively limited meaning with respect to the original Neolithic dimensions of the enclosures. This preservation bias must be recognized when attempting correlations between Neolithic enclosures in NW Europe using these metrics^1,4^: the older the enclosure in alluvial settings, the more likely it is to have been mostly eroded away.

Based on the curved shape of the Neolithic trenches exhumed by archaeological research in Noyen-sur-Seine, it is reasonable to assume that the Neolithic enclosures were originally subcircular and probably closed. Other Neolithic examples exist that would corroborate this hypothesis^1,4^ (Fig. 2). Outside of valleys, enclosures that are protected from alluvial river erosion are often circular, as evidenced by examples in England, Germany (e.g., Beusterburg and Windmill Hill^48,49^) or in the Somme region^50^ (Fig. 2a). In alluvial settings, at La Villeneuve-au-Châtelot (20 km upstream of Noyen-sur-Seine), it is only through the synthesis of 50 years of research—and the opening of numerous gravel pits from 1969 to 2021—that the full size of a monumental system of enclosures, delimiting an oval area of ca. 500,000 m^2^, could be envisaged^51^ (Fig. 2b). In Noyen-sur-Seine, assuming a circular or oval shape would imply an original size of approximately 90,000 m^2^ for the smallest enclosure (B). However, this assertion would need to be verified by excavating other pieces of the enclosure or associated trench. This would be difficult at Noyen-sur-Seine since the inferred northern limit of the enclosure—if preserved—would be beneath the hamlet of Port-Montain (Fig. 3). The largest enclosure (A) may have well been comparable to that of La Villeneuve-au-Châtelot. Downstream of Noyen-sur-Seine, numerous other enclosures with a semicircular shape were found in Balloy, Châtenay-sur-Seine, Gravon, Grisy-sur-Seine and Marolles-sur-Seine (Fig. 2). They were also thought to be built along a paleochannel-Seine River^1^. Based on the results of this study, we suggest that they most likely have the same preservation bias as in Noyen-sur-Seine, and that the edges of these enclosures correspond to erosional boundaries. Systematic dating of abandoned alluvial channels in their surroundings should make it possible to differentiate between the channels that were contemporaneous with these edifices and those that postdated and eroded them.

Last, we show that, apart from an abandoned paleochannel towards the SE part of the Neolithic enclosures, there are no traces of an active Neolithic Seine River in their vicinity. As discussed earlier, the most probable explanation is that the river’s main channel was located farther north due to an avulsion. This strongly indicates that the Neolithic populations were established in the alluvial plain but they avoided proximity to the river, contrary to earlier suggestions^32^. Hence, Neolithic populations most likely lived along relict paleochannels rather than active channels, i.e., not too far from the rivers but at a reasonable distance from floods. A comparable configuration was found for the Neolithic city of Mari, in Mesopotamia along the Euphrates River. There, intensive excavations conducted for more than 50 years by Margueron^52,53^ showed that the early site that was investigated for decades was only a small fraction (i.e., one third) of the original city, which was much larger. Geomorphological surveys conducted at the same time with the excavations of the city indicated that the city was built 2–3 km away from the Euphrates River so that it was never flooded, and it was suggested that a derivation canal was used to supply water to the city. Margueron and his team of geomorphologists demonstrated a circular shape for the city limits, which were largely eroded away by river activity afterwards^52,54^. These emblematic cases of Neolithic sites, the town of Mari on the Euphrates River and the enclosure of Noyen-sur-Seine (Fig. 2) clearly illustrate that the complete conservation of Neolithic enclosures is the exception rather than the norm in alluvial environments. These results should encourage a more systematic use of regional geomorphological investigations in archeology, in particular via the dating of paleochannels. Indeed, the palimpsest of alluvial traces (channels from different periods) must be subjected to a diachronic analysis to reconstruct the contemporary paleo-landscape of the Neolithic occupations. Ultimately, this geo-archaeological approach makes it possible to address the question of how these environments were perceived and managed by ancient societies given that the sedimentary and archaeological archives are systematically incomplete, most often detrimental over time.

## Methodology

### Migration

The calculations of the historical migrations rates of the paleo-course of the Seine River was based on two exceptional maps. The first one is a cadastral map of Noyen-sur-Seine in 1875, which belongs to the *Plan d’intendance* set, established by Louis Berthier de Sauvigny between 1777 and 1789 (https://archives.seine-et-marne.fr/fr/plans-dintendance). The second one is a navigation map from the *Administration des ponts et chaussées* of 1848, which was established before the major modification of the Seine River in 1845–1848 under the direction of Jacques Henri Chanoine (https://archiseine.metis.upmc.fr/). The two maps were georeferenced based on reference points that are still observed on the current cadaster (e.g., road intersections, buildings, castle, garden, etc.). In total, 27 reference points were used with uncertainties for position ranging from 4 to 40 m with an average of 18 m. The right and left bank were manually calculated for the 1785 and 1848 paths of the Seine River using the ArcGIS software.

To compute the channel migration, we used the approach developed by Lemay et al.^38^, which is based on the dynamic time warping algorithm^36^ (the Github repository is available here http://github.com/martin-lemay/ChannelPy.git). This algorithm calculates the similarities based on proximity along curved centrelines to calculate migration distances. We performed analyses on the left and right banks of the Seine River in between 1785 and 1848. Only the left bank is shown on Fig. 4a. The centreline points were resampled every 20 m. The maximum distance for the calculation was set to 240 m, i.e., approximately three times the width of the Seine River in these maps. As a result, the algorithm did not calculate the migration distance for the 1809 cutoff to the north of Noyen-sur-Seine. The distribution of the whole migration distances is shown in Fig. 4b. The fit for the migration distribution for the left and right banks is shown for comparison. The migration rates were computed by dividing the migration distances by the time span between the two maps.

### Radiocarbon ages

Field sampling to carry out radiocarbon dating of the abandoned channel sediment infill was conducted using a hand auger, or a peat borer in the organic-rich levels. The main lithologies (i.e., grainsize) were described directly in the field in the form of sedimentary logs. The cross-section of the f channel was constructed by interpolation between the sedimentary logs. Radiocarbon dating was performed on organic sediment or plant found along these logs in two laboratories: Beta Analytic Inc. in the USA and Poznan Radiocarbon Laboratory in Poland.

### Supplementary Information

Supplementary Information 1.

Supplementary Information 2.

## Data Availability

The datasets used and/or analyzed during the current study are available from the corresponding author upon reasonable request.
