# Supplementary figures and images for: Lateral river erosion impacts the preservation of Neolithic enclosures in alluvial plains
Source: Sci Rep. 2023 Oct 2;13:16566. doi: 10.1038/s41598-023-43849-6 (PMC10545758; doi:10.1038/s41598-023-43849-6)

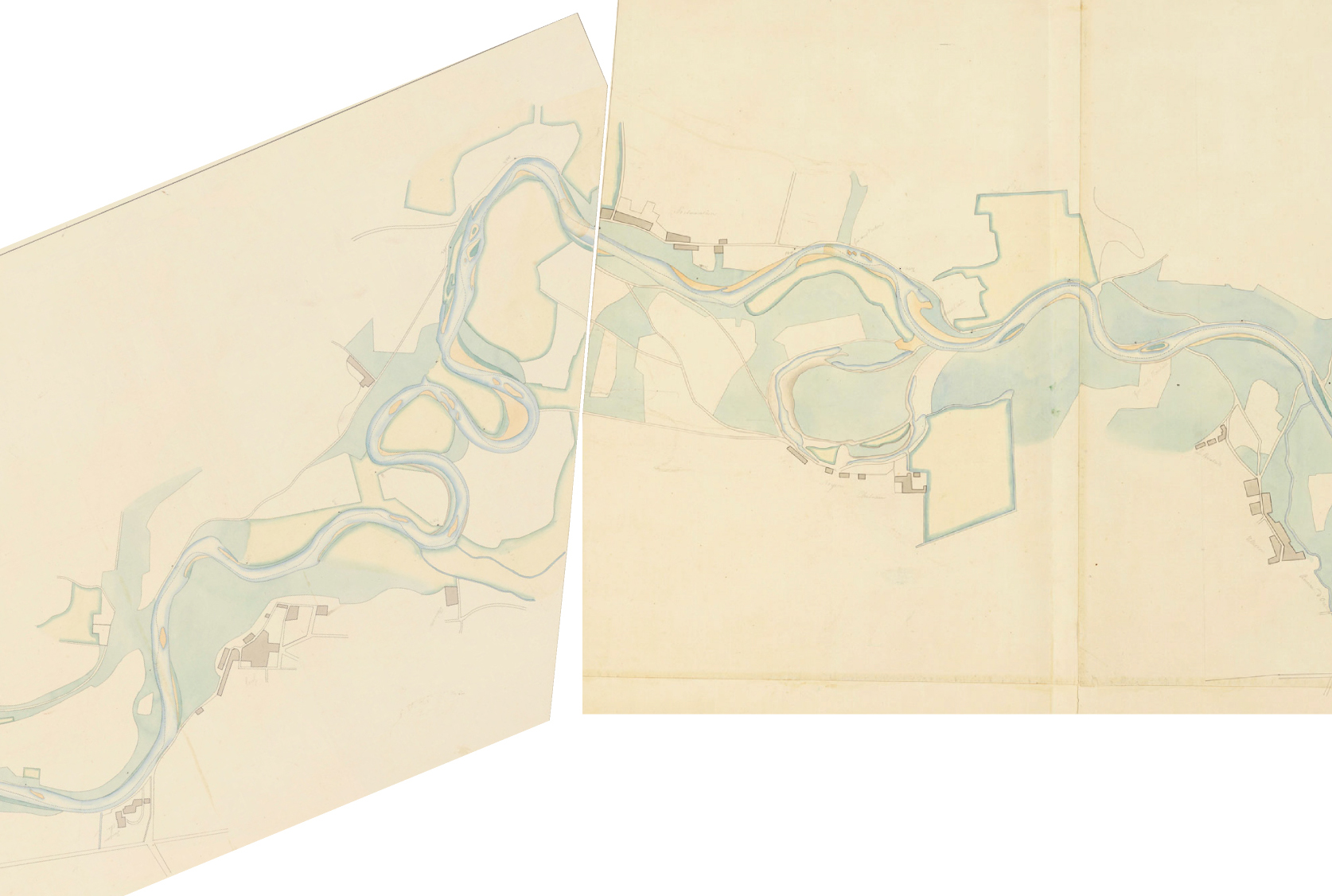

Supplement: Supplementary file 1 — Supplementary Information 1. [file 41598_2023_43849_MOESM1_ESM.jpg]

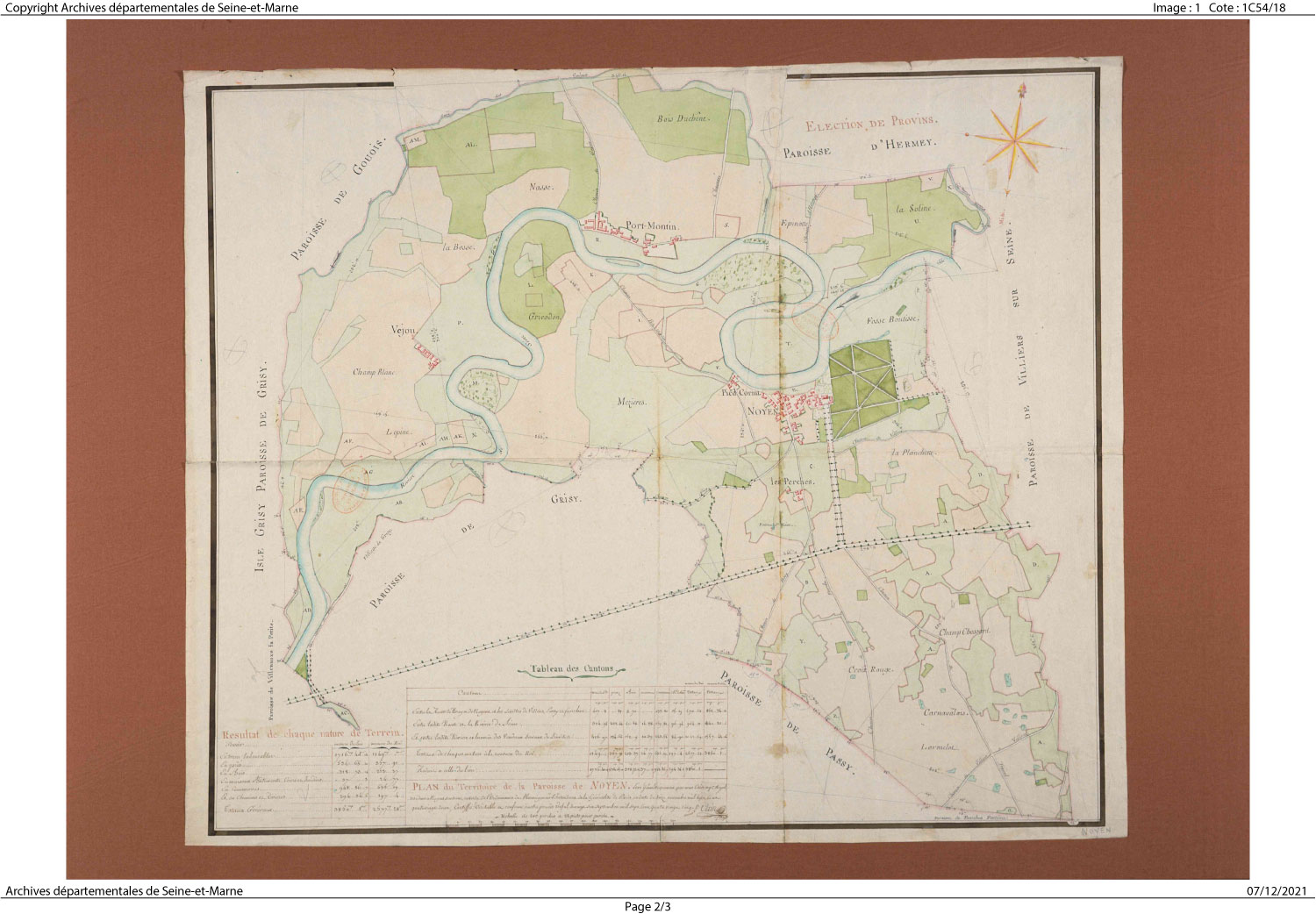

Supplement: Supplementary file 2 — Supplementary Information 2. [file 41598_2023_43849_MOESM2_ESM.jpg]
